# Extraction, Purification, Structural Characteristics, Biological Activities, and Applications of Polysaccharides from *Gastrodia elata*: A Review

**DOI:** 10.3390/molecules30020262

**Published:** 2025-01-10

**Authors:** Yan Yang, Yonglin Hai, Yongcheng Yang, Rouyuan Wen, Le Wang, Yan Qian, Jiaxian Zhang, Yongguo Li, Zhilong Shi, Haizhu Zhang

**Affiliations:** 1 College of Pharmacy, Dali University, Dali 671000, China; 17352095358@163.com (Y.Y.); hai520874@163.com (Y.H.); 15241821063@163.com (Y.Y.); 15828608051@163.com (R.W.); wl10192391@163.com (L.W.); 19188730787@163.com (Y.Q.); zhangjiaxian2023@163.com (J.Z.); 17787430793@163.com (Y.L.); 2 Yunnan Western Yunnan Medicinal and Edible Plant Resources Development Engineering Research Center, Dali 671000, China; 3 Yunnan Key Laboratory of Gastrodia and Fungi Symbiotic Biology, Zhaotong University, Zhaotong 657000, China

**Keywords:** *G. elata* polysaccharides, extraction, purification, structural characterization, biological activity

## Abstract

*Gastrodia elata* Blume is a well-known medicinal and edible plant in China, celebrated for its extensive history in traditional medicine and functional food applications. Among its key bioactive components, polysaccharides have drawn significant attention from researchers in the fields of health food and medicine due to their potential health benefits. Recent studies have revealed various biological activities associated with *G. elata* polysaccharides, including antioxidant, anti-tumor, anti-inflammatory, antibacterial, anti-aging, immune regulation, and neuroprotective properties. However, a comprehensive overview of these polysaccharides remains elusive. Specifically, relationship between the structure and activity of *G. elata* polysaccharides, along with the mechanisms through which various types exert their biological effects, has yet to be fully elucidated. This knowledge gap may impede the further development and utilization of *G. elata* polysaccharides in medicine, health products, food, and cosmetics. This paper provides a comprehensive overview of recent advancements in extraction, separation, purification, biological activities, and applications of *G. elata* polysaccharides. Additionally, it delves into structure-activity relationships and pharmacological mechanisms of these polysaccharides, giving support for future research to enhance their application in medicine, food, health products, and cosmetics.

## 1. Introduction

**Figure 1:**
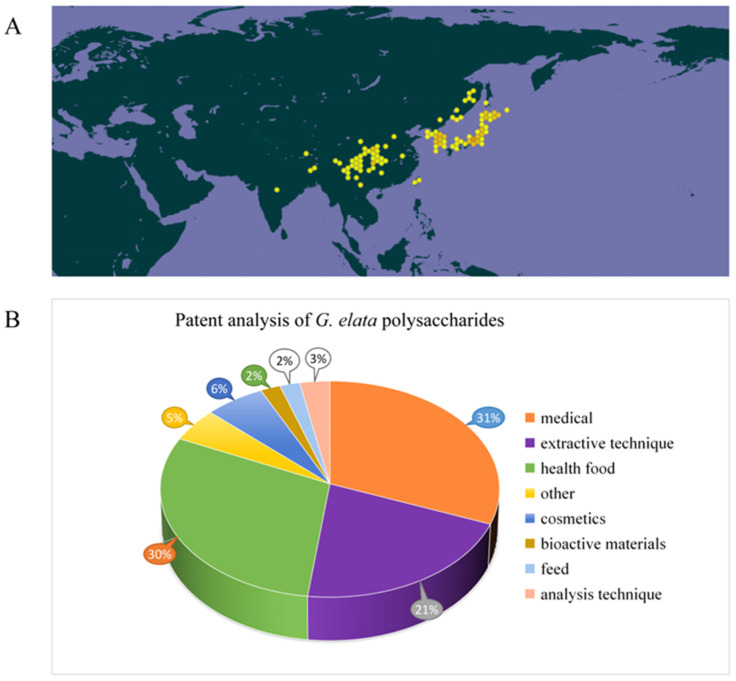
The distribution of *G. elata* in the world (https://www.gbif.org/) (accessed on 15 October 2024.) (**A**) and analysis of related patents on *G. elata* polysaccharides (**B**).

Gastrodiae Rhizoma, derived from the dried tuber of *Gastrodia elata* Bl., is a traditional Chinese medicine material known for its calming, antispasmodic, and liver-regulating properties [1]. In clinical practice, it is mainly applied for treating diseases like headache, dizziness, memory loss, hypertension, and epilepsy [2,3,4,5,6]. Gastrodiae Rhizoma has a history of over 2000 years of application in China, first documented in the *Shennong* herbal Scripture [7,8]. Widely distributed in Yunnan, Guizhou, Hubei, Sichuan, Shanxi, and other regions of China (Figure 1A), and traditionally used for both medicine and dietary purposes in these regions for centuries [1,6]. Due to its therapeutic effect and non-toxicity, it is divided in the catalog of homologous drugs and foods, referring from the National Health Commission of the People’s Republic of China and the 2020 edition of *the Chinese Pharmacopoeia*. According to the characteristics, such as arrow stem color, of *G. elata*, it can be divided into five types: *G. elata* f. glauca, *G. elata* f. elata, *G. elata* f.alba, *G. elata* f. elata, and *G. elata* f. viridis [9]. In these types, *G. elata* f. glauca and *G. elata* f. elata are widely used for artificial cultivation. At present, many drugs containing *G. elata* as the main raw material have been developed. Among them, more than 500 kinds of drugs, such as gastrodin injection, Tianma headache tablets, Tianma capsules, and Tianma tablets, received approval from the State Food and Drug Administration of China for listing [10]. In addition, *G. elata* is also an important raw material for food and health products. There are about 120 kinds of health foods made from *G. elata* and its extracts, which have the effects of improving sleep, assisting in lowering blood pressure, and regulating immunity [11]. With people’s pursuit of health, *G. elata* products are increasingly favored by consumers. Due to its unique growth mode, wild *G. elata* resources are becoming increasingly scarce. In China, *G. elata* has emerged as a significant economic crop in Yunnan, Guizhou, Sichuan, and Hubei provinces to fulfill consumer demand [12].

*G. elata*’s biological activity shows close correlation to its phytochemicals. Recent research has noted that chemical compounds isolated and identified from *G. elata* primarily consist of phenols [13], organic acids, polysaccharides [14], and steroids. These components confer *G. elata* with various pharmacological properties, including neuroprotection, cardiac protection, vascular regulation, antidepressant effects, anti-cancer activity, sleep enhancement, anticonvulsant properties, anti-inflammatory effects, and analgesic properties [15,16,17,18]. Among them, polysaccharides, as primary active ingredients within *G. elata*, possess various pharmacological activities, including anti-cancer [19], anti-virus, anti-oxidation, immune regulation, neuroprotection, and cardiovascular system regulation [20,21]. The chemical structure of *G. elata* polysaccharide is distinguished by a diverse monosaccharide composition. Its diverse physical and chemical properties, along with the associated health benefits, have garnered significant attention from researchers. Currently, more than 50 types of *G. elata* polysaccharides have been identified, including GaE-B, GaE-R, GEP-3, GEP-4, WGEW, and AGEW [14,22,23,24]. Predominantly, *G. elata* polysaccharide is classified as a glucan, primarily comprising an α-d-1,4-glycosidic bond as its main chain, potentially supplemented by α-1,3-glycosidic bonds and α-1,4,6-glycosidic bonds. Despite numerous studies focusing on the biological activity and structural characteristics of *G. elata* polysaccharide, its full potential remains underexplored and warrants further investigation for practical applications [25,26,27]. In addition, studies have found that polysaccharide structure shows close relation to biological activities [28]. However, only a little research has concentrated on the relationship between structural properties and biological activities of *G. elata* polysaccharides. In-depth research on *G. elata* polysaccharide is helpful for understanding various biological activities, as well as searching the relation between its function and structure. The patent analysis related to *G. elata* polysaccharides is shown in Figure 1B, mainly focusing on pharmaceuticals, health products, extraction technology, and other aspects (Figure 1B).

*G. elata* polysaccharide, as a natural bioactive component, holds significant market potential and development opportunities. However, there is still a lack of systematic and comprehensive summary of these polysaccharides, which may limit the development and utilization of *G. elata* polysaccharides in medicine, health care products, food, and cosmetics. This review seeks to provide a comprehensive summary of the existing literature on *G. elata* polysaccharides, covering extracting and purifying techniques, evaluating their respective strengths and limitations, and examining their molecular weight, monosaccharide composition, structural features, structure–activity relationships, and biological activities. Special emphasis is placed on exploring the mechanisms underlying the antitumor, immune regulation, antioxidant, and neuroprotective properties of *G. elata* polysaccharides; the review delves into potential challenges and future application prospects in *G. elata* polysaccharide research, offering valuable insights for its development and clinical use (this review collected the literature related to *Gastrodia elata* polysaccharide in the past 15 years).

## 2. Extracting and Purifying Techniques of *G. elata* Polysaccharides

### 2.1. Extraction and Separation of G. elata Polysaccharides

**Figure 2:**
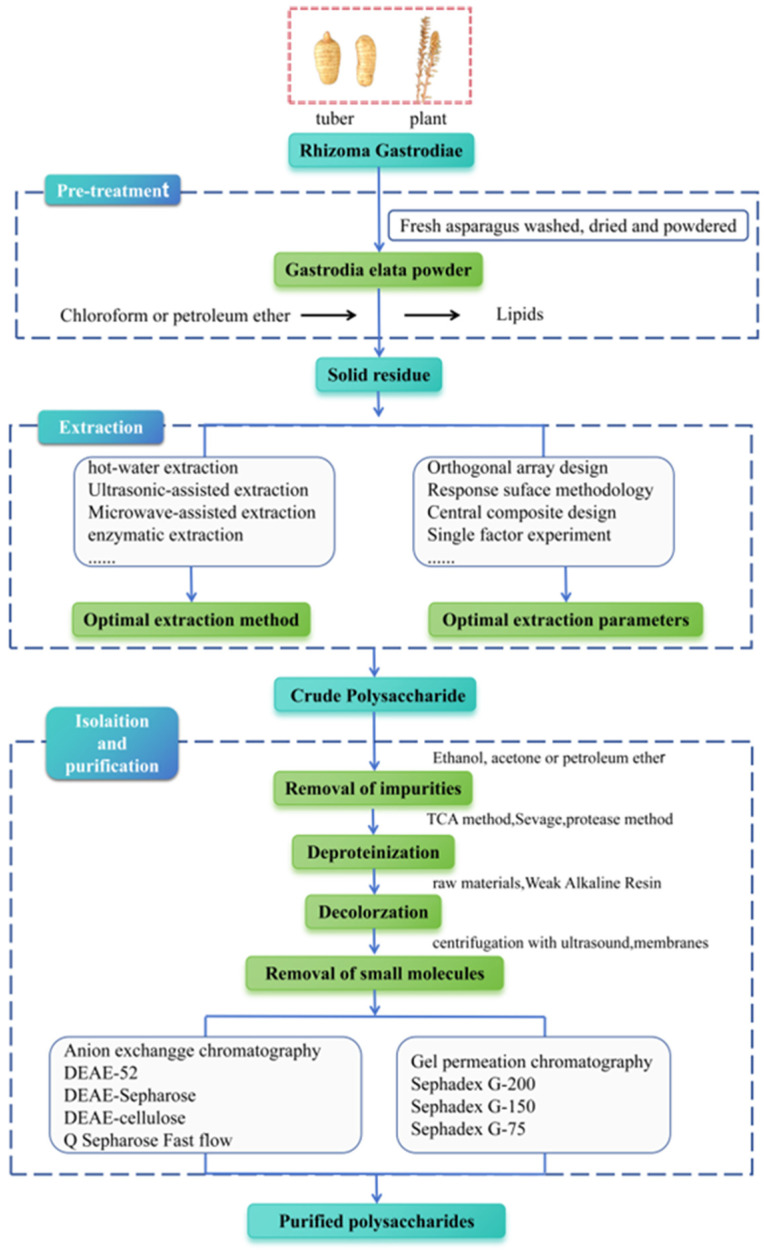
Schematic representation of extraction, isolation, and purification of *G. elata* polysaccharides.

Extracting plant polysaccharides is crucial for subsequent purifying processes, structural analysis, and investigation on pharmacological activities. Therefore, present optimizing extraction techniques for *G. elata* polysaccharides have become a primary research focus, with methods such as extracting by hot water, ultrasonic, microwave, and enzyme being commonly used [20,29]. The extraction and purification process is shown in Figure 2. Initially, organic solvents like chloroform and petroleum ether are utilized for degreasing to eliminate fat-soluble components, followed by specific extraction methods to extract polysaccharides from *G. elata*. Among them, the water extraction and alcohol precipitating method is the most extensively applied traditional extraction method for *G. elata* polysaccharide research [14,24,30,31]. The polarity of polysaccharides is high, and most types of polysaccharides have a large and stable solubility in hot water, while their solubility in organic solvents is small; therefore, hot water is generally applied as an extraction solvent. Moreover, the penetration of hot water into plant tissue is relatively strong, which can improve the extraction efficiency, making it more economical and safer to be used in production. However, this method is cumbersome to operate, and extraction by hot water at high temperature brings polysaccharide degradation, resulting in a relatively low extraction rate.

Innovative extraction methods, such as enzymatic hydrolysis, offer milder conditions for polysaccharide extraction. Enzymes can effectively break cell walls down, enhance cell permeability, and facilitate the dissolution of intracellular contents. This straightforward process ensures a high extraction rate [32,33]. Moreover, during the extraction of traditional Chinese medicine, impurities such as starch, protein, and pectin can compromise the quality of the extract. Appropriate enzymes (cellulase, pectinase, protease, etc.) can remove these impurities through gentle enzymatic hydrolysis reactions, thus improving the clarity of the extract. Ultrasonic-assisted extraction involves the usage of ultrasonic energy to mechanically destroy the cell wall structure of *G. elata*, leveraging thermal effects to enhance cell component dissolution. This method provides several advantages over others, including reduced extraction time, greater efficiency of extraction, and optimal preservation of polysaccharide activity, making it a favorable approach for polysaccharide extraction [20,34,35] Microwave-assisted extraction involves the usage of microwave energy to induce friction in plant polar molecules, leading to cell wall rupture and release of contents [36,37]. Wang et al. [38] conducted a study on *G. elata* using microwave-assisted extraction at 500 W for 120 s, with a material-to-liquid ratio of 1:40, a temperature of 70℃, and a 30-min extraction time, resulting in a 10.40% extraction rate of *G. elata* polysaccharide. Li et al. [39] observed *G. elata* polysaccharide’s extraction rate was 6.86% under 500 W, with a solid–liquid ratio of 1:40, and microwave extraction for 120 s at 70 °C. *G. elata* polysaccharide’s extraction rate obtained by microwave-assisted extraction is low. Notably, a novel methodology utilizing ionic liquid ultrasound-assisted extraction for *G. elata* polysaccharides has been introduced. Ionic liquids offer several advantages over traditional organic solvents, including low toxicity, volatility, and flammability; effective performance with both non-polar and polar components; recyclability; superior solubility; environmental friendliness; and excellent chemical stability.

### 2.2. Purification of G. elata Polysaccharides

Separation and purification of polysaccharides are necessary processes to study polysaccharides. The process typically involves separation, purification, and identification of purity. This task is particularly challenging in carbohydrate research due to the presence of impurities, such as pigments, proteins, and oligosaccharides, within crude polysaccharides [40,41]. Because of these impurities, the biological activity of polysaccharides is often affected, which also poses great difficulties for qualitative and quantitative analyses and structural determination of polysaccharides. Moreover, excessive impurities hinder the assessment of the activity–structure relation of polysaccharides. Thus, isolating and purifying crude polysaccharides is essential for obtaining homogeneous samples and accurately determining their structural characteristics. Currently, the main methods used for polysaccharide purification are deproteinization, decolorization, fractionation precipitation, cellulose column chromatography, salting out, quaternary ammonium salt precipitation, ion exchange column chromatography, ultrafiltration, preparative high-performance liquid chromatography, cellulose acetate membrane filtration, gel column chromatography, affinity chromatography, etc. [40].

Following extraction of *G. elata* polysaccharides, the resultant mixture contains a variety of impurities, including polar impurities such as fatty acids. These weakly polar impurities take advantage of the characteristics of polysaccharides to be insoluble in organic reagents. By using ethanol (≥80%), acetone, or petroleum ether for repeated precipitation and washing, these weakly polar impurities can be eliminated [42]. Furthermore, crude polysaccharides typically contain impurities like proteins, pigments, and small molecules, which can negatively affect both their purity and biological activity. Therefore, it is imperative to extract crude polysaccharides from *G. elata* for subsequent separation and purification, as outlined in Table 1. Currently, proteins in crude polysaccharide of *G. elata* can be effectively removed using trichloroacetic acid, Sevag [14,30,43,44,45] (n-butanol: chloroform is 1:5 or 1:4), or protease [46] methods. Among them, the trichloroacetic acid method has a strong effect but can lead to polysaccharide degradation. The Sevag method is milder and better preserves polysaccharide integrity, although its protein removal efficiency is lower, typically requiring multiple repetitions to achieve satisfactory results. The protease method also shows good efficacy, but it may disrupt the peptide chains on glycoprotein and affect the biological activity of polysaccharide. To enhance the purification of crude polysaccharides from *G. elata*, an effective approach involves combining an improved protease method (papain or trypsin) degradation with the Sevag method for protein removal. This strategy minimizes the need for multiple organic solvent washes, thereby reducing the possibility of polysaccharide loss during gel precipitation. Activated carbon is typically utilized to adsorb pigment substances in crude *G. elata*; however, it may also inadvertently adsorb some polysaccharides, leading to their loss. The polysaccharides of *G. elata* may contain phenolic components, which may darken the color. This type of pigment is not captured by activated carbon but can be effectively adsorbed by weak alkaline resin. Small molecules, such as inorganic ions and oligosaccharides, can be eliminated through processes such as dialysis, ultrasonic centrifugation, or ultrafiltration with specialized membranes. Furthermore, homogeneous polysaccharides extracted from *G. elata* were primarily obtained through gel and cellulose anion exchange column chromatography. Generally, these methods exhibit distinct separation characteristics, with chromatography of ion-exchange being suitable for neutral or acidic polysaccharide separation [47]. Gel filtration chromatography using Sephadex G-200, Sephadex G-100, and Sephadex G-50 is extensively applied as chromatographic media to separate polysaccharides with different molecular weights.

## 3. Structural Characteristics of *G. elata* Polysaccharide

**Figure 3:**
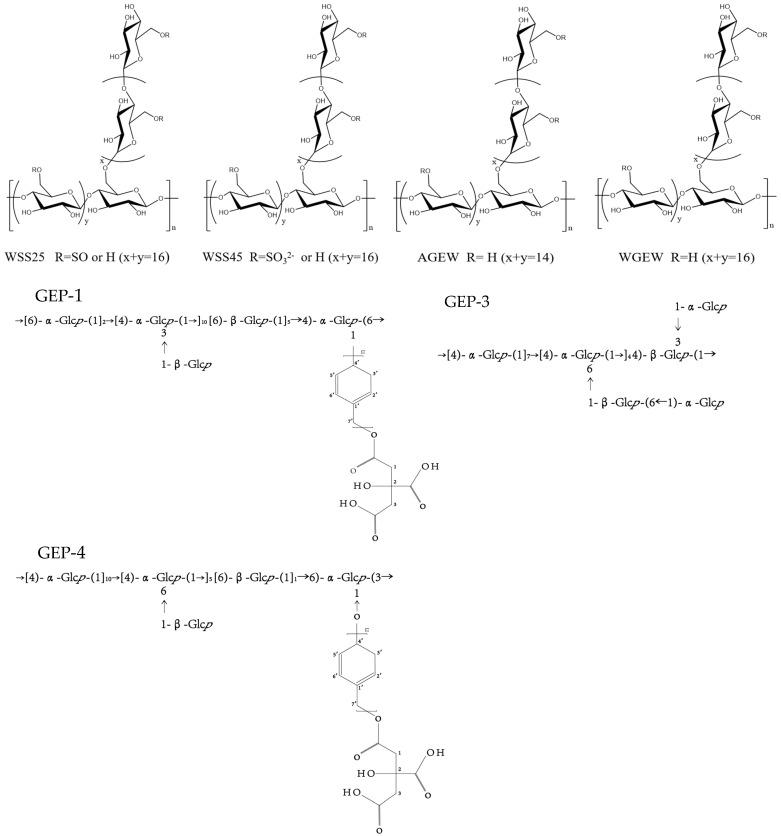
Polysaccharide structure of WSS25, WSS45, AGEW, WGEW, CEP-4, GEP-3, and CEP-1.

The analysis of the structural characteristics of polysaccharides is crucial; polysaccharide structure diversity directly determines its biological activity [94]. Structural characteristics of polysaccharides were analyzed, including glycosidic bond type, connection mode, high-order structural conformation, relative molecular mass, and monosaccharide composition, which are key factors in determining the structure and function of polysaccharides [95]. The monosaccharide composition of *G. elata* polysaccharides is diverse, featuring over 50 identified types, including GaE-B, GaE-R, GEP-3, GEP-4, WGEW, and AGEW (Table 2, Figure 3). The polysaccharides derived from *G. elata* Blume are primarily composed of glucose, with their sugar chains predominantly structured as α-(1,4) pyran-d-glucan [96]. The homogeneous polysaccharide GES extracted from *G. elata* by Li et al. [49] was found to have a molecular weight of 292.596 kDa and a monosaccharide composition ratio of Glu:Gal:GalA:Arabinose:Fru = 88.21: 4.48: 4.40: 0.87: 0.85 Ning et al. [30] extracted four varieties of GaE polysaccharides: GaE-B (*G. elata* Bl. f. glauca S. chow polysaccharide), GaE-R (*G. elata* Bl. f. elata polysaccharide), GaE-G (*G. elata* Bl. f. viridis Makino polysaccharide), and GaE-Hyb (hybridization of *G. elata* Bl. f. glauca S. chow and *G. elata* Bl. f. elata polysaccharides). These polysaccharides share the same monosaccharide composition, primarily consisting of glucose, xylose, and mannose, although the content varies significantly among the different varieties. Huo et al. [14] discovered that GEP-3 is a 1,4-glucan with a molecular weight of 20 kDa. They also identified the homogeneous polysaccharide GEP-4, with a molecular weight of 25 kDa, which features a complex main chain structure. This marks the first time this new polysaccharide has been obtained and characterized. Chen et al. [45] successfully isolated a water-soluble polysaccharide (GEP2-6) of 2.7 × 10^6^ Da from G. elata, achieving a purity of 99.2%. Spectral and chemical analyses indicated that GEP2-6 is a glucan composed of α-(2→6) and α-(1→4) glycosidic bonds. Enzymatic hydrolysis further characterized GEP2-6’s local structure, showing the presence of α-1,6-Glcp, β-1,4,6-Glcp, α-1-Glcp, and α-1,4-Glcp in a molar ratio of 1.32:1.08:0.93:31.27. Zhu et al. [52] separated a homogeneous polysaccharide, PGE, from *G. elata* by water extraction and Sephadex G-200 separation and purification. Structural analysis revealed that the *G. elata* polysaccharide was primarily composed of glucose, rhamnose, and mannose in small amounts, with an average relative molecular mass (Mr) of 1.54 × 10^3^ kDa. The main chain is composed of α-(1,4)-d-glucopyranose with 1,3- and 1,4,6-branched glucopyranose. The *G. elata* polysaccharide PGEB-3H, as obtained by Ming et al. [97], was primarily composed of glucose with a mean molecular weight of 28.8 kDa and a specific optical rotation of +206.3°. It featured a main chain of α-(1,4)-d-glucopyranose and included 1,4- and 1,4,6-connected branches. Chen et al. isolated the *G. elata* polysaccharide WTMA through processes comprising degreasing, extraction by water, precipitation by alcohol, deproteinization, dialysis, and DEAE column separation. Structural analysis revealed that WTMA consists solely of glucose, with a mean molecular weight of 7.0 × 102 kDa, a specific optical rotation of +382°, and α-(1,4)-glucan as the main chain. Zhu et al. [20] summarized that *G. elata* polysaccharide mainly consisted of glucose and mostly contained α-1,4-glucan, α-1,4,6-glucan, and α-1,3-glucan. Chen et al. [55] extracted *G. elata* polysaccharides through water extraction, alcohol precipitation, protein removal, freeze-drying, and additional steps. Structural analysis revealed that the polysaccharide consisted of glucose, with a mean molecular weight of 8.75 × 10^3^ kDa and a polysaccharide content of 91.3%. The *G. elata* polysaccharide isolated by Liu et al. [98] primarily consisted of glucose, as well as a small amount of mannose, xylose, and arabinose. Qiu et al. [23] isolated two homogeneous α-d-glucans, known as water-extracted polysaccharides WGEW and alkali-extracted polysaccharide AGEW. The average molecular weight of WGEW was 100 kDa, with a specific rotation of +92°. AGEW had an average molecular weight of 280 kDa and a specific rotation of +166.6°. Both WGEW and AGEW were composed of glucose monosaccharides, with the main chain structure as α-d-(1,4)-glucan. They also contained terminal glucose (T-Gle) and glucose linked in 1,4 and 1,4,6 configurations, present in varying molar ratios. Hong et al. [93] utilized a combination of chemical methods, such as sugar composition and methylation analyses, alongside spectroscopic techniques like 13C-NMR and IR, to comprehensively identify the structures of seven homogeneous polysaccharides: GBI-1, GBI-2, GBI-3, GBII, GBIII, GBIV, and GBV. The findings revealed that GBI-1’s main chain comprised α-d-1,4-linked glucose with occasional branches at the 6-position and had a molecular weight of 14600. In contrast, GBI-2 and GBI-3 had molecular weights of 8700 kDa and 7000 kDa, respectively. The authors suggested that, despite the differing molecular weights, GBI-1, GBI-2, and GBI-3 exhibit identical structures and may represent distinct degradation fragments of the same homogeneous polysaccharide. GBII was identified as a glucan comprising 27 glucose units with a molecular weight of 4300, featuring a main chain of α-d-1,4-linked glucoses with branches at the 6-position and some glucuronic acid. GBIII was characterized as a 19,000 molecular weight pectin-type polysaccharide, while GBIV was with a molecular weight of 87,000. Lastly, GBV was identified as a sulfated polysaccharide with a molecular weight of 100,000 and a sulfate content of 25%. The sugars in GBV consisted of galactose and glucose at a molar ratio of 43:57.

## 4. Biological Activity of *G. elata* Polysaccharide

**Figure 4:**
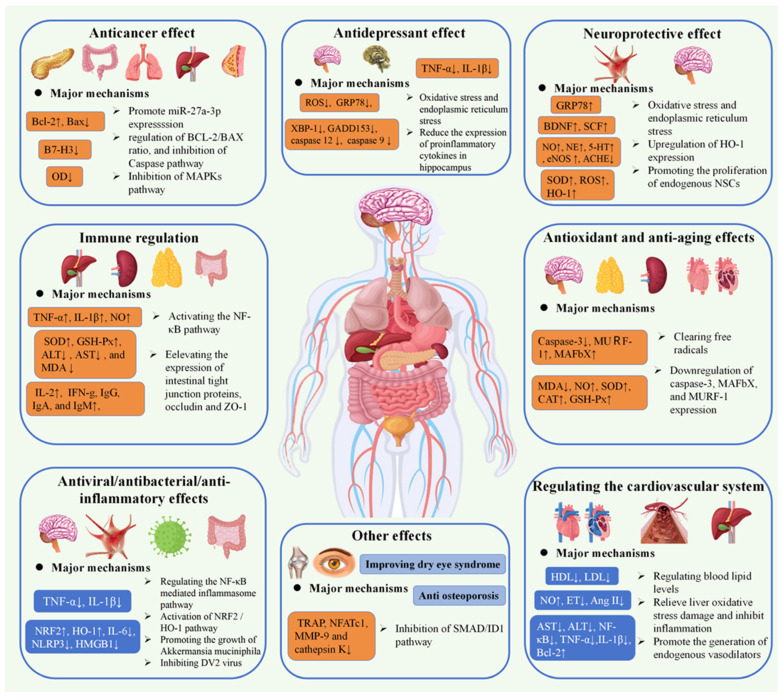
Schematic representation for the bioactivities of *G. elata* polysaccharides (↑: It indicates that receptors, hormones, proteins, etc. are up-regulated or increased. ↓: It indicates that receptors, hormones, proteins, etc. are down-regulated or reduction).

*G. elata* is a promising edible and medicinal plant with a variety of bioactive components, which has great value for application in the field of medicine and food. *G. elata* exhibits a variety of biological functions of polysaccharides, comprising anti-oxidation, treatment of cardiovascular system diseases, anti-tumor effects, neuroprotection, immune regulation, anti-inflammatory properties, and so on. Both mushroom polysaccharides and *G. elata* polysaccharides exhibit immune-enhancing effects. Mushroom polysaccharides are known to stimulate macrophages and enhance cytokine secretion, thereby activating the immune response, while *G. elata* polysaccharides enhance immune balance by modulating the proportion and function of T cell subsets. The precise regulation of lymphocytes by *G. elata* polysaccharides is particularly significant in the context of immune disorders [101,102]. Furthermore, both *Ganodermalucidum* (*G.lucidum*) and *G. elata* polysaccharides demonstrate anti-tumor activity. *G. lucidum* polysaccharides inhibit the signal transduction pathways of tumor cells, whereas *G. elata* polysaccharides act directly on tumor cells and bolster immune surveillance [103,104]. The potential of *G. elata* polysaccharides in preventing tumor recurrence and metastasis is noteworthy. Additionally, *Lycium barbarum* polysaccharides and *G. elata* polysaccharides exhibit antioxidant properties. *Lycium barbarum* polysaccharides function by scavenging free radicals to protect cells, while *G. elata* polysaccharides enhance cellular defense by increasing antioxidant enzyme activity. Particularly noteworthy is the ability of *G. elata* polysaccharides to provide significant protection to nerve cells against oxidative damage, a crucial aspect in the prevention and treatment of neurodegenerative conditions [105,106]. The similarities and distinctive characteristics in the biological activity of *G. elata* polysaccharides and other natural polysaccharides provide valuable resources for medicine and health care, contributing to the advancement of human health. Table 3 provides a comprehensive overview of the biological activities associated with polysaccharides from *G. elata*, and Figure 4 illustrates the pharmacological activities and mechanisms of action associated with *G. elata* polysaccharides.

### 4.1. Anti-Cancer Activity

An increasing amount of research indicates that polysaccharides extracted from *G. elata* exhibit potent anti-cancer properties. Laboratory studies have demonstrated that polysaccharides extracted from *G. elata* (WTMA) possess the ability to suppress PANC-1 cancer cell proliferation. The products of acid hydrolysis WTMA-AD-O and WTMA-AD-I have significant anti-tumor activity [22]. Specifically, an increase in the molecular size of homogeneous polysaccharides leads to a significant decrease in the growth activity of anti-cancer cells. Furthermore, *G. elata* polysaccharides (PGEs) have been found to hinder MCF-7 cell growth by inducing late apoptosis and blocking the G2/M phase, with homogeneous polysaccharide PGE-40 exhibiting a notably high suppression rate [107]. PGE-40 has the highest inhibition rate, which may be attributed to its spherical conformation, molar mass distribution, and dense structure. Notably, acetylated and sulfated *G. elata* polysaccharides (AcGEP and SGEP) have better anti-breast cancer activity [19]. For rat glioma cells (C6), sulfated *G. elata* polysaccharides (GEPs) could significantly inhibit the growth and migration of glioma cells [108]. This enhancement in activity could potentially be attributed to the structural alterations in GEP that occur during the modification process [90]. In addition, suppressive effects by *G. elata* polysaccharides on pheochromocytoma were mainly achieved by increasing the ratio of BCL-2/BAX protein and suppressing caspase-related pathways activation [79]. For transplanted tumors, *G. elata* polysaccharides can induce inflammatory infiltration to impede tumor cell proliferation and enhance the immune response in tumor-bearing mice [109]. *G. elata* polysaccharide can also suppress the growth and invasion of colon cancer cells under arsenic exposure and promote their apoptosis. The underlying mechanism may be associated with the promotion of miR-27a-3p expression and the inhibition of B7-H3 expression [46].

### 4.2. Immunomodulatory Activity

Research has shown that *G. elata* polysaccharides exhibit significant immunomodulatory effects. *G. elata* polysaccharides (GEP-1) can elevate IL-1β, NO, and TNF-α in macrophages. Experimental evidence supports that its immune regulatory effects are primarily mediated through NF-κB pathway activation [43]. GEP-2, another polysaccharide from *G. elata*, has been found to decrease TNF-α and IL-1 levels, enhance T and B lymphocytes’ growth, and consequently exhibit immune regulatory properties [115]. Moreover, GEPs have demonstrated the ability to elevate serum levels of IL-2, TNF-α, IFN-γ, IgG, IgA, and IgM, as well as spleen and thymus indices in cyclophosphamide-induced immunosuppressed mice dose-dependently [48,114]. Selenium-enriched *G. elata* polysaccharides (Se-GEP) have been shown to enhance the body weight and feed intake in immunosuppressed mice, increase spleen and thymus indices, improve phagocytic function, and simultaneously activate Th1 and Th2 lymphocytes, resulting in elevated secretion levels of NO and TNF-α [51]. Compared with the GEP group, high selenium changed the structure of *G. elata* polysaccharide, thereby enhancing the immunomodulatory activity [51].

### 4.3. Antioxidant/Anti-Aging Activity

In vitro research demonstrated that polysaccharides extracted from aboveground parts of diverse *G. elata* varieties possess antioxidant properties. The effectiveness of these polysaccharides in reducing and scavenging DPPH and ABTS free radicals increases with higher concentrations of polysaccharides. Among the above-ground polysaccharides of *G. elata* f. viridis, the DPPH free radical scavenging rate was found to be the highest at the same concentration of polysaccharide solution [35]. The homogeneous polysaccharides GEP-1 and GEP-2, isolated from *G. elata*, exhibited a strong DPPH scavenging rate and high inhibition rates for superoxide anion and hydroxyl radical [74]. Zhang et al. [62] and Li et al. [60] utilized the DPPH method and Fenton system to evaluate the scavenging efficiency of DPPH free radicals and hydroxyl radicals in *G. elata* polysaccharides. The results indicated that *G. elata* polysaccharides showed significant scavenging effects on both DPPH and hydroxyl radicals, with concentration-dependent scavenging ability. Furthermore, *G. elata* polysaccharides were found to enhance the learning and memory abilities of d-galactose-induced aging mice and increase SOD activity in the brains of aging mice, elevating GSH-Px activity in blood, reducing MAO activity in brain tissue, and decreasing MDA levels, thereby promoting brain nerve tissue recovery [116]. Selenized *G. elata* polysaccharides demonstrated higher clearance rates for DPPH and ABTS+ free radicals compared to regular *G. elata* polysaccharides, with a maximum iron reduction of 0.99% ± 0.24%. [82]. Structural characterization analysis showed that SeGEP had been successfully accomplished, and SeGEP had reduced the particle size, increased the absolute value of Zeta potential, and improved the stability in solution by comparing it with GEP [82].

### 4.4. Antiviral/Antibacterial/Anti-Inflammatory Activity

#### 4.4.1. Anti-Inflammatory Activity

HO-1 can directly regulate the expression of proinflammatory factors; furthermore, HO-1 can catalyze the decomposition of heme to generate CO, which can inhibit the expression of the classical inflammatory regulator NF-κB, thereby inhibiting the proinflammatory signal. *G. elata* neutral polysaccharide (NPGE) can downregulate IL-1β, NLRP3, IL-6, HMGB1, and TNF-α and upregulate NRF2 and HO-1 expressions. It also facilitates translocation of NRF2 to the nucleus and suppresses neuroinflammation [118]. Additionally, *G. elata* polysaccharides (GBP) can mitigate the increase in proinflammatory cytokine levels induced by vincristine (Vin). GBP treatment results in decreased IL-6, IL-8, TNF-α, and IL-1 β mRNA levels within the spinal cord, sciatic nerve, and DRG [99]. Simultaneously, *G. elata* polysaccharide also alleviates inflammatory injury by regulating the NF-κB-mediated inflammasome pathway and reducing TNF-α and IL-1β, the proinflammatory factors [76].

#### 4.4.2. Antibacterial Activity/Improvement of Intestinal Flora

GEP-3 was 1,4-glucan, GEP-4 comprising a backbone of →[4)-α-Glc*p*-(1]_10_→[4)-α-Glcp-(1→]_5_[6)-β-Glc*p*-(1]11→6)-α-Glcp-(3→ and two branches of β-Glc_p_ and p-hydroxybenzyl alcohol citrate, with repeating p-hydroxybenzyl alcohol attached to the backbone chain at O-6 position of →4,6)-α-Glc*p*-(1→ and O-1 position of →3,6)-α-Glc*p*-(1→. GEP-3 and GEP-4, the homogeneous polysaccharides isolated from *G. elata*, can notably facilitate Akkermansia muciniphila growth and also promote the myxomycetes in the fecal microbiota of high-fat diets (HFD) [14]. GEP-1 was composed of →[6)-α-Glc*p*-(1→]_2_[4)-α-Glc*p*-(1→]_10_[6)-β-Glcp-(1]_5_→4)-α-Glc*p*-(6→, with three branches of β-Glc*p* and CA-repeating p-HA attached to the backbone chain at the O-3 position of 1,3,6-linked α-Glc*p* and the O-1 position of 1,4,6-linked α-Glc*p*. Bioactivity tests showed that GEP-1 could promote the growth of *A. muciniphila* and *L. paracasei* strains [24,44]. Additionally, *G. elata* polysaccharide (GBP) also restored the imbalance of intestinal microbiota by increasing the levels of Firmicutes, Ligilactobacillus, and Bacteroidetes Alloprevotella, while simultaneously reducing the levels of Proteobacteria-Escherichia coli-Shigella [31].

#### 4.4.3. Antiviral Activity

The structures of two glucans, WGEW and AGEW, isolated from *Gastrodia elata* Bl. Their structures were deduced as an α-d-(1→4)-glucan. Their sulfate derivatives with distinct degrees of substitution (DS) were prepared. All sulfated derivatives showed strong anti-dengue virus bioactivities. The structure–activity relationships (SAR) between the polysaccharides and their sulfated derivatives were also investigated. Results showed that the higher the DS is, the more potent the impact on the dengue virus infection would be [23,56]. Moreover, WSS45 has been found to effectively inhibit DV2 infection in BHK cells, primarily by disrupting viral adsorption during the early stages of the viral life cycle. WSS45 also enhances the detachment of viruses from cell surfaces in BHK cell lines, effectively inhibiting dengue virus serotype (DV2) by interfering with the interaction between viruses and their target cells [119].

### 4.5. Neuroprotective Effects

Multiple studies have confirmed the neuroprotective effects of polysaccharides derived from *G. elata.* For instance, the *G. elata* polysaccharide GEP can partially reverse the pathological changes induced by corticosterone (Cort) in a depression model using PC12 cells, thereby providing neuronal cell protection. Alongside significant apoptosis in PC12 cells, the expression level of GRP78 also increased, demonstrating a clear dose–response relationship [79,113,130]. Research involving the administration of *G. elata* polysaccharides (PGB) to rats with focal cerebral ischemia demonstrated that PGB could provide neuroprotective effects by upregulating BDNF and self-consistent field expression [53]. Moreover, *G. elata* polysaccharides (GRPS) can enhance the memory of young rats with cerebral palsy, with the corresponding mechanism related to increases in contents of NO, NE, and 5-HT in the cerebral cortex and hippocampus, with reduction in ACHE and increase in eNOS expression, thus protecting hippocampal tissue [122]. Using *G. elata* polysaccharides (GBP) and glutamate (Glu) to co-incubate HT22 hippocampal neurons from mice, researchers have discovered that GBP can reduce Glu-induced damage in HT22 neurons. At the same time, GBP was found to increase the SOD activity and ROS clearance ability of HT22 cells dose-dependently and time-dependently, indicating that the protective effect of GBP on HT22 cells may be linked to its enhanced antioxidant capabilities [123]. The combination of electroacupuncture and *G. elata* polysaccharides can significantly increase Nestin and BDNF expressions in the CA3 area of ischemic hippocampus from rats with cerebral ischemia, suggesting that it may exert a protective effect on neurons in the ischemic area by promoting endogenous NSCs proliferation [124,125]. After treatment for cerebral palsy, it was found that *G. elata* polysaccharide could reduce the apoptosis of brain tissue in cerebral palsy rats, playing an effective neuroprotective role [126].

### 4.6. Treatment of Cardiovascular Diseases

Purified *G. elata* acidic polysaccharides were mainly composed of xylose, glucose, galacturonic acid, and glucuronic acid. The *G. elatas* acidic polysaccharide fraction significantly reduced systolic blood pressure in SHR on a high-fat diet. In addition, acidic polysaccharides positively regulated lipid levels in SHR [57]. Meanwhile, *G. elata* acid polysaccharide also inhibits atherosclerosis by downregulating low-density lipoprotein and total cholesterol levels, thereby regulating the blood lipids of Sprague Dawley rats (SD) [127]. In addition, *G. elata* polysaccharides inhibited liver cell apoptosis by upregulating anti-apoptotic factor Bcl-2 and inhibiting Bax protein expression. Upregulating the Nrf2/GPx signaling pathway helps alleviate oxidative stress damage, protect, and delay the symptoms of non-alcoholic fatty liver disease (NAFLD) induced by HFD, exhibiting a significant liver protective effect [76]. PGE was composed of glucose, with an average molecular weight of 1.54 × 10^3^ kDa. The structure of PGE was 1→3 and 1→4,6-branched-glucopyranose that had a linear backbone of (1→4)-linked-d-glucopyranose (Glcp). ACE-inhibitory activity results showed that PGE was efficient in inhibiting ACE, and the IC50 value was 0.66 mg/mL [52,128].

### 4.7. Other Activities

Zhou et al. established a depression model using PC12 cells and observed a protective effect by *G. elata* polysaccharides (GEP) on cortisol-induced apoptosis in PC12 cells, suggesting their potential as a treatment for depression [113]. Furthermore, *G. elata* polysaccharides (GEPs) have demonstrated the ability to ameliorate lipopolysaccharide (LPS)-induced depressive behavior in mice, with their potential mechanism associated with reduction in cytokines TNF-α and IL-1β mRNA expression in the hippocampus and improving hippocampal neuronal function [121]. Chen et al. discovered that sulfated polysaccharide (WSS25) suppresses osteoclast formation in RANKL-induced mice by impeding the SMAD/ID1 pathway in the treatment of osteoporosis. This was demonstrated by the establishment of an ovariectomized ICR albino mouse model [112]. A phospholipid complex derived from *G. elata* polysaccharides significantly enhances tear volume, extends tear film rupture time, and improves the regenerative capacity of corneal epithelial cells, particularly in terms of tear film stability [79].

## 5. Structure–Activity Relation in *G. elata* Polysaccharide

In the activity–structure relation of *G. elata* polysaccharides, the connection between structure and antitumor activity is extensively researched. Chen et al. discovered that the biological activity of three *G. elata* polysaccharides (WTMA, WTMAE-AD-I, and WTMA-AD-O) is influenced by their molecular size. Specifically, an increase in the molecular size of homogeneous polysaccharides brings a significant drop in the growth activity of anti-cancer cells. Dai et al. [107] observed that *G. elata* polysaccharides (PGES) can suppress MCF-7 cell growth by facilitating late apoptosis and the blockage in the G2/M phase. Among these, PGE-40 exhibited the highest inhibition rate, likely due to its spherical conformation, molecular weight distribution, and compact structure. However, further investigation is needed to fully understand the relationship between the structural characteristics of PGEs and their antitumor efficacy. Dai et al. [131] found that the inhibitory rate of ultrasound-extracted *G. elata* polysaccharides (PGE) on MCF-7 cell proliferation was generally higher than that of PGE extracted by hot water. This could be due to the lower molecular weight of the PGE extracted by ultrasound compared to that extracted by hot water. Polysaccharides with appropriate molecular weights tend to exhibit higher anti-tumor activity. The spherical structure of PGE may have a greater impact on inhibiting MCF-7 cell proliferation compared to the loose hyperbranched structure, making it more effective in inhibiting MCF-7 cell growth. Cheng et al. [88] found that high-molecular-weight *G. elata* polysaccharides were discovered to exhibit a more potent suppressive effect on the in vitro proliferation of tumor cells, specifically on cancer cells HepG2 and Hela.

Apart from that, the relationship between the structure and immunomodulatory activity of *G. elata* polysaccharides has been extensively researched. GEP-3 was discovered by Huo et al. [14], and GEP-1, discovered by Guan et al. [43], belongs to α-(1→4)-glucan. The key difference lies in the molecular weights, with GEP-3 at 20 kDa and GEP-1 at 76 kDa, indicating a higher degree of polymerization in GEP-1. The molecular weight of polysaccharides is crucial for their activity, and substitution level in branched chains also plays a significant role in immune activity [132]. The substitution level of GEP-1 is less than that of GEP-3, which also indicates that there may be a gap in their immune activity. Chen et al. [56] found that the homogeneous polysaccharide RGP-1b exhibited stronger immune activity compared to RGP-1a, possibly attributed to the varying monosaccharide compositions and structures of the two polysaccharides. Nonetheless, additional investigation is required to fully understand the biological activity and structural characteristics of both RGP-1a and -1b.

Structural modification of *G. elata* polysaccharides may improve their original activity or generate new activity. For example, Liu et al. [19] found improved anti-breast cancer activity by *G. elata* polysaccharide following structural modifications, such as sulfation and acetylation, especially for acetylated GEP. This enhancement in activity could potentially be attributed to the structural alterations in GEP that occur during the modification process. Dou [80] sulfated the hydroxyl group of *G. elata* polysaccharide (SYGEP) and acetylated it (AcYGEP). The findings indicated that the chain conformation of SYGEP was predominantly rigid, irregular, and highly branched, while the chain conformation of AcYGEP was more spherical. The effect of *G. elata* polysaccharide derivatives on the inhibition of MCF-7 cell proliferation was assessed using the MTT method. The results demonstrated that both modifications enhanced the anti-breast cancer activity of the *G. elata* polysaccharide, with AcYGEP exhibiting superior activity, possibly due to its specific chain conformation. Ma et al. [51] found that compared with the GEP group, high selenium-modified *G. elata* polysaccharides (Se-GEP) enhanced the proliferation, phagocytosis, and secretion of NO and interleukin-1β in RAW264.7 cells, thereby enhancing the activity of immune regulation. In addition, Wen et al. [82] found that selenization modification can enhance the antioxidant capacity of *G. elata* polysaccharides. Zhou et al. [130] found that sulfated *G. elata* polysaccharide (GEPS) had a certain protective effect on PC12 cell injury induced by corticosterone (CORT), but it was not significantly improved compared to *G. elata* polysaccharide (GEP), and other biological activities need to be further studied. Qiu et al. [23] identified sulfated derivatives of *G. elata* polysaccharides with varying degrees of substitution (DS), attributing the substitution position to O-6 based on the (13) C NMR spectrum. These derivatives exhibited significant activity against the dengue virus. Research on the structure–activity relationship revealed that an increased DS is associated with a stronger impact on dengue virus infection.

Our research indicates that the chemical composition and structural characteristics of polysaccharides are critical to their biological activities. Grasping the connection between structure and function is vital for the study of polysaccharides. However, investigations into the structure-function relationship of *G. elata* polysaccharides remain scarce and lack comprehensive analysis. This limitation largely arises from the intricate chemical composition and structure of polysaccharides, which complicates the assessment of their higher-order structures. To further investigate the connection between the structure and biological activity of *G. elata* polysaccharides, innovative ideas and methodologies are essential for analyzing their advanced structures in future studies. This approach will not only enhance our understanding of the conformational relationships of these polysaccharides but also offer a more solid theoretical foundation for drug development, disease treatment, and various applications of polysaccharides. Consequently, the exploration of novel techniques for analyzing the higher-order structures of *G. elata* polysaccharides will be a key priority for future research, paving the way for a deeper understanding of their true conformational relationships.

## 6. Application of *G. elata* Polysaccharide

**Figure 5:**
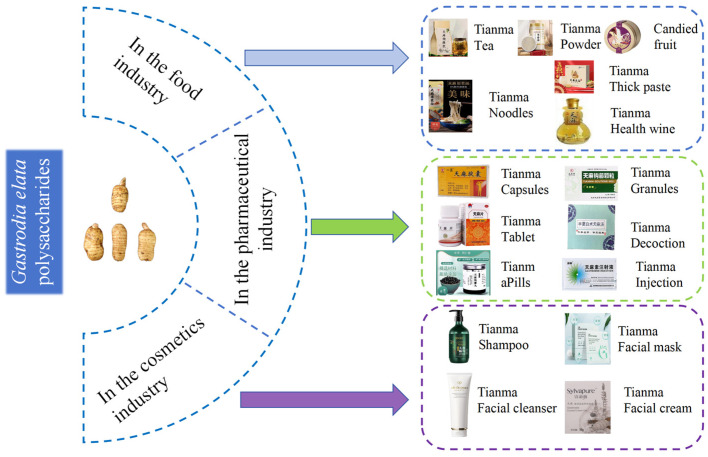
Practical and potential applications of *G. elata* polysaccharides.

*G. elata* polysaccharides are mainly used in the fields of pharmaceuticals, health foods, and cosmetics, as shown in Figure 5.

### 6.1. In the Food Industry

*G. elata* is increasingly being used as a health food in folk medicine. Dishes and foods containing *G. elata* have various health benefits, such as tonifying blood, regulating blood pressure, enhancing immunity, promoting calmness, and improving sleep [11]. Despite its long history of use as a health food in traditional medicine, *G. elata* is not widely approved for use in the national special food information query platform (http://ypzsx.gsxt.gov.cn/specialfood/#/food) (accessed on 10 october 2024.). Only 120 approved health foods are currently available, with 39 products utilizing *G. elata* extract as the main ingredient. The remaining products are derived from *G. elata* [1]. These health foods mainly focus on immune regulation, sleep improvement, anti-aging, blood pressure control, anti-hypoxia, and memory enhancement. They come in various forms, including liquor, hard capsules, tea, tablets, oral liquid, and granules. Hard capsules are the most common form, with 68 different varieties [133,134].

### 6.2. In the Pharmaceutical Industry

Polysaccharides are the primary active components found in *G. elata*. An increasing number of medications are incorporating *G. elata* or its extract as key ingredients. According to the State Food and Drug Administration (https://www.nmpa.gov.cn/) (accessed on 20 September 2024), these medications include *G. elata* pills, tablets, and capsules. These compound preparations are known for their ability to dispel wind, remove dampness, clear collaterals, relieve pain, and tonify the liver and kidneys. They are commonly used to treat conditions such as memory loss, insomnia, and headaches, and their efficacy is ensured through the establishment of quality standards [8]. For people with low immune function, such as the elderly or long-term patients, *G. elata* polysaccharide may help to restore and improve immune levels. *G. elata* polysaccharide can effectively scavenge free radicals in vivo and reduce the damage of oxidative stress to cells. For example, it can delay cell senescence and protect tissues and organs from oxidative damage. *G. elata* polysaccharide helps to improve insulin resistance and regulate blood glucose levels. For example, in patients with pre-diabetes, it may help prevent the occurrence of diabetes. Combined with hypoglycemic drugs, it may improve the effect of diabetes treatment. *G. elata* polysaccharide has a protective effect on the nervous system, which may help prevent and treat neurodegenerative diseases, such as Parkinson’s disease and Alzheimer’s disease.Alleviate the symptoms caused by nerve injury, such as improving neurological function after cerebral ischemia–reperfusion injury. It should be noted that although *G. elata* polysaccharide has a variety of potential applications, it is still in the research and development stage, and its specific application effect and safety need further clinical trials and verification.

### 6.3. In the Cosmetics Industry

With in-depth research on *G. elata* polysaccharides, they have significant practical value and promising application prospects in daily cosmetics. Its natural, safe, and functional characteristics provide unique advantages for cosmetics. In recent years, the concept of “plant-based skincare” has gained increasing praise and popularity among consumers. *G. elata*, a traditional Chinese medicinal herb, extract has been included in the “Catalogue of Cosmetic Raw Materials (2021 Edition)”. Products with mid- to high-end representation, mainly composed of *G. elata*, are rarely marketed. Gastrodin, polysaccharides, flavonoids, and amino acids in *G. elata* have the effect of replenishing water and moisturizing, which provides a preliminary material and functional basis for the application of *G. elata* in cosmetics. Currently, only a few products, such as Shiliwei *G. elata* cleansing cream, Lifu Queen *G. elata* herbal moisturizing mask, *G. elata* polysaccharide moisturizing cream, and *G. elata* Pleuropterus multiflorus shampoo, are available in the market. Research on *G. elata* has primarily focused on its medical and edible value, with its potential applications in cosmetics warranting further exploration [135]. The polysaccharide found in *G. elata* offers distinct advantages as a moisturizing agent, thanks to its abundance of hydrophilic hydroxyl structures that exhibit hygroscopic, moisturizing, high-viscosity, and film-forming properties. It can play a role in nourishing water, moisturizing, improving skin function, and moisturizing the stratum corneum in cosmetic skincare formulations. Simultaneously, it can eliminate free radicals from the skin, promote metabolism, and contribute to anti-wrinkle, whitening, and overall skin function improvement [136].

## 7. Conclusions and Future Prospects

As a traditional Chinese medicine sourced from food, *G. elata* is widely utilized in the fields of medicine, health food, and cosmetics. Polysaccharides represent key bioactive constituents in *G. elata*, exhibiting pharmacological activities such as antioxidant effects, anti-tumor properties, immunomodulation, and memory enhancement. The pharmacological activity of *G. elata* polysaccharides is closely related to their relative molecular mass, monosaccharide composition, glycosidic linkages, and other structural features. Structurally modified selenated *G. elata* polysaccharides have been shown to enhance antioxidant capacity. An increase in the molecular size of homogeneous polysaccharides results in a significant decrease in anti-cancer cell growth activity. Among these, α-(1→4)-glucan represents the most important class of *G. elata* polysaccharides, contributing to the restoration of the human immune system. Furthermore, *G. elata* polysaccharides are abundant in glucose, xylose, and mannose, which can stimulate the immune system, activate the immunoregulatory network, and enhance immune function.

The extraction, isolation, and purification of *G. elatas* polysaccharides form the foundation for subsequent research and applications. Currently, the primary methods for extracting *G. elatas* polysaccharides encompass water extraction, alcohol extraction, ultrasound-assisted extraction, and other traditional techniques. With advancements in modern technology and the ongoing development of polysaccharide research, innovative methods such as microwave-assisted extraction, ionic liquid-assisted extraction, and supercritical fluid extraction are gaining traction for their application in polysaccharide research.

In conclusion, the various beneficial effects of aspalathus polysaccharides have generated significant interest in this area of research. This study systematically summarizes the existing literature on the extraction, purification, structural properties, bioactivity, and conformational relationships of *G. elatas* polysaccharides, along with an exploration of their potential future applications. Despite the increasing number of studies on *G. elatas* polysaccharides, investigations are still in their nascent stages, and numerous challenges remain unresolved. Future investigations should prioritize optimizing extraction processes and enhancing purification methods while focusing on the interplay between the structure and function of *G. elatas* polysaccharides, as well as elucidating their mechanisms of action. Such efforts will further expand their applications in the fields of food, nutraceuticals, and pharmaceuticals. This paper lays a scientific foundation for advancing the application of *G. elatas* polysaccharides, which are anticipated to possess significant market potential and promising prospects in medicine and functional foods.

## Figures and Tables

**Table 1:** Extraction and purification methods of polysaccharides from *G. elata*.

Extraction							Purification		
Polysaccharide Fraction	Extraction Method	Time	Temperature (°C)	Solid-Liquid Ratio (g/mL)	Power/Enzyme	Total Production (%)	Polysaccharide Fraction	Purification Method	Ref.
GEPs	Hot water extraction	2 h		1:10		58.4		Deproteinization using Sevag method; the crude polysaccharides were decolorized by using the D101macroporous resin	[48]
GaE-B GaE-R GaE-Hyb GaE-G	Water extraction	4 h	90	1:20		GaE-B: 5.67 GaE-R: 4.21 GaE-Hyb: 6.63 GaE-G: 7.82		Sevag reagent to remove the associated protein; Dialysed in a dialysis bag with flowing distilled water	[30]
GBP	Water extraction and ethanol precipitation methods	1 h	60	1:30				Deproteinization by Sevag method; dialysis was performed using a 3500 Da dialysis membrane.	[31]
	Ultrasonic Assisted Extraction	1 h	70	1:30	160 w			Wash three times with ethanol and acetone, centrifuge for 10 min and collect the sediment.	[49]
GEP-3; GEP-4	Hot water extraction	4 h	70	1:30			GEP-3; GEP-4	Precipitation and deproteinisation using the Sevage method; DEAE-cellulose column; Sephacryl 200 column; 5 kDa membrane separation equipment.	[14]
PGE-20, PGE-40, and PGE-120	Ultrasonic assisted extraction with deionized water	20, 40, and 120 min	70	1:30	200 w		PGE-20, PGE-40, and PGE-120	Add 95% ethanol and leave overnight at 4 °C for polysaccharide precipitation.	[19]
GEP-1	Water extraction and alcohol precipitation					11.11	GEP-1	Separate using a 10 kDa membrane separation device and elute with water.	[24]
GEP-D GEP-1	Hot water extraction					GEP-D: 0.89 GEP-1: 0.67	GEP-D GEP-1	Sevag solvent for protein removal; DEAE-Sepharose Fast Flow column; Sephacryl S-400HR column.	[43]
GEP	water extraction	4 h	100	1:15			GEP	Removing proteins using Sevag method.	[50]
GEP	water extraction	4 h					GEP	Ethanol precipitation, deproteinised by Sevag method, dialysed in 3000 Da dialysis bag.	[45]
SGEP AcGEP	Ultrasonic assisted extraction with deionized water	30 min	70	1:30	200 w			95% ethanol precipitation.	[19]
	Hot water extraction	1 h		1:10				Precipitation and washing with anhydrous ethanol and acetone followed by chloroform/butanol to remove proteins.	[45]
GEP, GEP-SA, GEP-Cy5.5	Boiling water extraction	2 h		1:30			GEP, GEP-SA, GEP-Cy5.5	Precipitate with anhydrous ethanol and remove protein 6–7 times with Sevag reagent; DEAE-52 cellulose.	[50]
	Ultrasonic extraction with distilled wate	36 min	56	1:59				Anhydrous ethanol precipitation and Sevag method for deproteinization.	[35]
Se-GEP	Boiling water reflux extraction						Se-GEP	Protein removal by Sevag method; ethanol precipitation and centrifugation; use of DEAE-52 ion exchange resin columns.	[51]
PGE	Water extraction	2	60	1:40		15.81	PGE	Sepag method for deproteinization, Wash crude polysaccharides on Sephadex G-200 column, further purify with ultrafiltration tube.	[52]
PGB	Water extraction							DEAE-52 cellulose col umn (2.6 × 50 cm) chromatography separation and purification for the preparation of PGB.	[53]
WGEW AGEW	Extract with boiling water	4 h				WGEW: 11.76 AGEW: 18	WGEW AGEW	DEAE cellulose column elution.	[23]
GPs	Water extractionr	4 h					GPs	Proteins were removed with 15% trichloroacetic acid, and then a column with DEAE-52 cellulose eluted the crude polysaccharides.	[54]
GEP	Thermostatic water bath extraction	4 h	90	1:10			GEP	Protein removal by Sevag method; Precipitate in ethanol and filter through Whatman GF/A filter paper.	[55]
WTMA	Boiling-water extraction	4 h				WTMA: 0.69%	WTMA	Proteins were removed with trichloroacetic acid; the supernatant was dialysed; ethanol precipitation yielded crude polysaccharides, which were then eluted on a DEAE cellulose column.	[22]
RGP-1a RGP-1b		66 min	74	1:54		6.11	RGP-1a RGP-1b	SProtein removal by eaveg method, purification by DEAE cellulose-52 and Sephadex G-100 elution.	[56]
Crude polysaccharides fraction Acidic polysaccharide fraction	Boiling water extraction	3 h	60	1:10		Crude polysaccharides fraction: 2.47 Acidic polysaccharide fraction: 0.61	Crude polysaccharides fraction Acidic polysaccharide fraction	The crude polysaccharide extract was further purified using ion exchange chromatography on DEAE Sepharose CL-6B.	[57]
	Extraction with petroleum ether reflux	2 h		1:30				Proteins were removed by the Seaveg method, decolourised by 1% activated carbon and the filtrate was left overnight; filtered and dried.	[58]
	Add 80% ethanol for water bath reflux	2 h	60	1∶8				Dehydrated ethanol precipitation of polysaccharides.	[59]
	Hot water extraction	30 min	95	1:20				Proteins were removed with lead acetate, ethanol precipitated and then processed by elution on a DEAE-52 cellulose column.	[60]
	Boiling water extraction	4 h		1:75				Anhydrous ethanol for alcohol precipitation	[61]
	Hot water extractio	45 min	65	1:40		3.28		Sevag method for deproteinization and alcohol precipitation drying.	[62]
	Ultrasonic assisted extraction of citrate buffer solution	30 min	50	1:40	300 w			Sevage method for deproteinization; Perform alcohol precipitation with 5 times the volume of methanol.	[63]
	Ultrasonic assisted water extraction	34 min	66	1:45				Decolorize with hydrogen peroxide, precipitate with 95% ethanol, and wash with ether, petroleum ether, and anhydrous ethanol respectively.	[64]
	Hot water extraction	2.5 h	70	1:37		22.38		Anhydrous ethanol for alcohol precipitation.	[65]
	Petroleum ether degreasing followed by pure water extraction	4 h	100	1:15		1.21		A mixture of chloroform and n-butanol was used for protein extraction and removal, and ethanol precipitation.	[33]
GEP, GEP II, GEPs, and GEP IIs	Water extraction and alcohol precipitation method							DEAE-52 elution separation, Sephadex G-100 elution separation.	[66]
SGCP2 SGCP3 NSGCP2 NSGCP3	Hot water extraction	31 min	67	1:16		SGCP2: 21.50 SGCP3: 26.65 NSGCP2: 10.94 NSGCP3: 10.28	SGCP2 SGCP3 NSGCP2 NSGCP3	Alcohol precipitation and protein removal by Sevag reagent, purification by dialysis, chromatography on DEAE-52 cellulose columns and Sephadex G-50 gel columns	[67]
PGEB-3-H	Pure water extraction	3 h	50				PGEB-3-H	Ethanol precipitation, deproteinisation, dialysis; further purification by DEAE-52 cellulose column chromatography and Sephadex G-100 column chromatography.	[68]
WPGB-A-H WPGB-A-L	Pure water extraction	3 h	50	1:10		WPGB-A-H: 0.824% WPGB-A-L	WPGB-A-H WPGB-A-L	Sevag method for deproteinization, dialysis, ethanol precipitation, DEAE-52 cellulose chromatography, and Sephadex G-100 chromatography for separation and purification.	[69]
	Water extraction and alcohol precipitation method	3 h	120	1:40				The protein was removed by Sevag method and separated by Sepharose 6 Fast Flow gel.	[70]
	Water extraction and alcohol precipitation method	3 h	120	1:40				Proteins are removed by the Seaveg method, decolourised by activated carbon, filtered and the filtrate left overnight.	[71]
GEP1 GEP2	Boiling water extraction	2 h	96			GEP1: 20.8 GEP2: 25.4	GEP1-G GEP2-G	Ethanol precipitation, Sevage method to remove protein, dialysis, purification.	[72]
GEP GEP1 GEP2	Pure water extraction	2.5 h	70			13.15	GEP GEP1 GEP2	95% ethanol precipitation, Sevag method for deproteinization, activated carbon decolorization, DEAE-52 cellulose chromography elution	[73]
	Pure water extraction	2 h	80	1:30		13.46		Anhydrous ethanol for alcohol precipitation	[74]
	Degreased with petroleum ether and extracted by reflux in a water bath	4 h	100	1:40				Deproteinised and precipitated. The precipitate was washed with anhydrous ethanol and acetone.	[75]
	Water bath reflux extraction	2 h	90	1:8				Anhydrous ethanol for alcohol precipitation.	[76]
	Water extraction and alcohol precipitation	1 h		1:30				Sevag method for deproteinization and retention of relative molecular weight 1000 in dialysis bags, dialyzed in water for 24 h.	[77]
	Water extraction and alcohol precipitation method	2 h	80	1:40		9.61		Dialysis using dialysis bags, alcohol precipitation and Sepharose 6-Fast Flow gel column chromatography elution.	[27]
	Pure water extraction	3 h	120	1:40				Protease degradation + sevag method for protein removal.	[78]
TM-20, TM-40, TM-60	Hot water extraction	3.1 h	90	1:32			TM-20, TM-40, TM-60	Ethanol fractional precipitation.	[79]
	Water bath reflux extraction	2 h	80					Ethanol precipitation, protein removal by enzymatic hydrolysis Sevag method, decolourisation with H2O2, dialysis with distilled water.	[46]
YGEP SGEP	Ultrasonic assisted hot water extraction	1 h	70	1:30				The polysaccharides were precipitated, washed, and then eluted by flow fractionation with an asymmetric flow field.	[80]
	Water extraction and alcohol precipitation	2 h	50					Sevag method for protein removal and activated carbon for pigment removal, DEAE cellulose column for elution.	[81]
GEP SeGEP	Hot water extraction	2 h	80	1:30			GEP SeGEP	Sevag reagent (chloroform: n-butanol = 4:1) was used to remove free proteins, followed by precipitation with anhydrous ethanol.	[82]
	Ultrasonic assisted water extraction	48 min	78	1:16		17. 22		Extracted with trichloromethane n-butanol and precipitated with ethanol. The residue was washed with ethanol, acetone and ether.	[83]
GBP-Ⅰ, GBP-Ⅱ	Hot water extraction	6 h					GBP-I, GBP-II	filtered, deproteinised by the Sevag method and washed repeatedly with anhydrous ethanol, acetone and ether.	[84]
	Water bath extraction method	4 h	100	1:30				Protein removal with chloroform n-butanol and alcohol precipitation with anhydrous ethanol.	[85]
	Water extraction and alcohol precipitation	2	60	1:40		15.81		Enzyme + Sevag method combined to remove proteins, and Sephadex G-200 combined with ultrafiltration tube for separation and purification.	[86]
GeB40-1 GeB80-4	Water extraction	36 min and 34 min	58 and 66	1:37 and 1:45		9.25, 32.78	GeB40-1 GeB80-4	Ethanol fractionation, papain + Sveg method for protein removal, dialysis method, DEAE-52 cellulose column chromatography.	[87]
WTM-1,WTM-2, WTM-3, WTM-4, WTM-5, WTM-6	Water extraction and alcohol precipitation	2 h	60	1:30		5.47	WTM-1,WTM-2, WTM-3, WTM-4, WTM-5, WTM-6	Protein removal by the Sevage method, removal of small molecule magazines and pigments by hollow fibre ultrafiltration membranes, separation and purification by DEAE-52 and Sephadex G-100.	[88]
WPGB-A-H	Water bath extraction	3 h	50	1:10		0.824		Sevag method for deproteinization, dialysis, ethanol precipitation, DEAE cellulose and Sephadex G-100 chromatographic separation and purification.	[89]
PRG1 PRG2 PRG3 PRG4 PRG5	Water extraction and alcohol precipitation	65 min	78	1:51				Colour was removed using activated charcoal, proteins were removed using the sevage method, dialysed using distilled water, precipitated with alcohol and further purified using cellulose DE-52 and gel G-100.	[90]
	Water extraction and alcohol precipitation, Ultrasound assisted alcohol extraction	3 h and 1 h	60, 50	1: 60				Degreased and decolorized with petroleum ether and anhydrous ethanol, precipitated with ethanol, removed protein by Sevage method, and dialyzed with deionized water.	[91]
TM1 TM2 TM3	Water extraction and alcohol precipitation	2 h	50				TM1 TM2 TM3	Sevag method for deproteinization, dialysis with distilled water, precipitated by ethanol, washed several times with organic reagents, and chromatographed on a DEAE (OH−) cellulose column (2.6 × 50 cm).	[92]
GB I-1 GB I-2 GB I-3 GB II GB III GB IV GB V	Boiling water extraction	6 h				GB I-1 GB I-2 GB I-3 GB II GB III GB IV GB V	GB I-1 GB I-2 GB I-3 GB II GBIII GBIV GB V	DEAE Sepharose Fast Flow ion exchange chromatography, SephadexG-10 column desalination, molecular sieve gel column chromatography elution, and then SephadexG-10 desalination.	[93]

**Table 2:** Name, molecular weights, monosaccharide composition, and structures of polysaccharides from *G. elata*.

Polysaccharide Fraction	Configuration	Molecular Weights/Molecular Mass	Monosaccharide Composition	Structural Characteristic	Analytical Techniques	Bioactivities	Ref.
GEPs		292.596 kDa	Glu:Gal:GalA:Ara:Fru = 88.21: 4.48: 4.40:0.87:0.85		HPGPC, MALLS, 1260 HPLC, HPAE-PAD	Strong immune-enhancing effects	[48]
GBP		53.43 ± 0.83	Glu:Fru = 8.7:1		GPC-MALLS-RI,HPLC, HPLC-RI.	Neuroprotective effects.	[99]
GEP-3 GEP-4		GEP-3: 20 kDa GEP-4: 25 kDa	Glu	GEP-3: 1,4-glucan GEP-4: →[4)-α-Glcp-(1]10→[4)-α-Glcp-(1→]5[6)-β-Glcp-(1]11→6)-α-Glcp-(3→ and two branches of β-Glcp and p-hydroxybenzyl alcohol citrate, with repeating p-hydroxybenzyl alcohol attached to the backbone chain at O-6 position of →4,6)-α-Glcp-(1→ and O-1 position of →3,6)-α-Glcp-(1→.	HPGPC, PMP-HPLC, LC/MS, FT-IR, NMR, SEM.	Promote the growth of *Akkermansia muciniphila*.	[14]
RGP-1a RGP-1b		RGP-1a: 19.25 kDa RGP-1b: 3.92 kDa	RGP-1a: Fru:Glu = 1:10.68 RGP-1b: Glu		HPLC-RID, HPGPC, FTIR.	Immunological activity.	[56]
GEP-1		20.15 kDa	Glu	The fully O-methylated GEP-1 was composed of 2,3,4,6-Me4-Glcp, 2,3,6-Me3-Glcp, 2,3,4-Me3-Glcp, 2,3-Me2-Glcp and 2,6-Me2-Glcp with a molar ratio of 2.23:9.69:4.93:1.08:2.07.	HPGPC, PMP-HPLC, LC/MS, FT-IR, NMR, SEM.	Promote the growth of *Akkermansia muciniphila*.	[24]
GEP-1	α	76.444 kDa	Glu:Gal:Ara:Man = 92.04:4.79:2.19:0.34	α-(1→4)-glucans.		Enhancing immune.	[43]
GEP		42.58 × 10^4^ Da	Glucose, Galactose and Galacturonic acid	α-glucopyranose structure.	HPGPC, HPIC, FT-IR.	Neuroprotective effects.	[100]
GEP SGEP AcGEP		GEP: 12.9 × 10^7^ g/mol SGEP: 9.3 × 10^7^ g/mol AcGEP: 9.3 × 10^7^ g/mol			FT-IR, AF4-MALS-dRI.	Anti-Breast Cancer Activity.	[19]
GEP2–6		2.7 × 10^6^ Da	Glu	GEP2–6 was a glucan linked by α-(1 → 4) and α-(1→6) glycosidic bonds.	HPGPC/SEC, UPW, HPLC-ELSD, FT-IR, GC-FID, NMR, AFM.	Antioxidant.	[45]
GEP		12 kDa	Glu:Gal = 82.33:1.0		High-performance ion exchange chromatography, FT-IR, UV, NMR.		[50]
*G. elata Bl. F. elata* *G. elata Bl. F. Glauca S Chow* *G. elata Bl. F. Viridls MalKino*			*G. elata Bl. F. elata:* Glu:Gal:GalUA:Man:Ara:Rha = 29.16:28.89:13.85:9.81:8.17:6.20 *G. elata Bl. F. Glauca S Chow*:Glu:Gal:GalUA:Man:Ara:Rha = 37.32:26.29:10.97:4.69:4.10:4.37 *G. elata Bl. F. Viridls MalKino*: Glu:Gal:GalUA:Man:Ara:Rha = 21.84:30.78:14.76:9.30:9.65:4.18		HPAEC.	Antioxidant activity.	[35]
GEP Se-GEP		GEP: 524.4 kDa Se-GEP: 488.9 kDa	Glu		UV, FT-IR, HPLC, SEM, EDX, HPGPC.		[51]
PGE		1.54 × 10^3^ kDa	Glu	PGE was composed of (1→4)-linked-glucose. Residues of branch structure might be (1→4,6)-linked-glucose, (1→3)-linked-glucose and T-glucose.	HPLC, UV, FT–IR, NMR, GC–MS.	Inhibiting ACE activity.	[52]
WGEW AGEW	α	WGEW: 1.0× 10^5^ AGEW: 2.8 × 10^5^	Glu	α-d-(1→4)-glucan with an α-(1→4) linked branch attached to O-6 branch points with different branch degrees.	GC, GC-MS, NMR.	Anti-dengue virus bioactivities.	[23].
GPs		2.71 × 10^5^ Da	Glu		HPSEC.	Immune modulating activities.	[54]
GEP		875.19 kDa	Glu		FT-IR, UV, LC-10A.	Antioxidant activity.	[55]
WTMA		7.0 × 10^5^ Da	Glu	α-1→4)-glucan with α-(1→4) linked branches attached to 0–6 at branch points.	HPGPC, GC, GC–MS, NMR, MALDI-TOF.	Antitumor.	[22]
PGEB-3H		28.8 kDa	Glu	PGEB-3H was mainly composed by glucose, and had a (1→4)-α -d-glucan main chain occasionally branched with α-1,6 glycosidic linkage.	PC, GC, HPGPC, IR, NMR.	Hypolipidemic Activity.	[97]
SGCP2 SGCP3 NSGCP2 NSGCP3		SGCP2: 31,027 Da SGCP3:727,650 Da NSGCP2: 39,812 Da NSGCP3: 39,991 Da	SGCP2: Man:Rha:GlcA:Glc:Ara = 1.99:1.72:3.60:70.68:22.01 SGCP3: Man: Rha:GlcA:Glc:Ara = 1.19:1.06:2.30:73.81:21.64 NSGCP2: Man:Rha:GlcA:Glc:Xyl:Ara = 2.96:1.39:3.08:70.05:0.63:21.88 NSGCP3: Man:Rha:GlcA:Glc:Ara = 0.86:0.99:2.03:74.47:21.65	SGCP2, SGCP3 and NSGCP2, NSGCP3 are both composed of three glycosidic bonds, namely: d-Glcp-1 →, → 4) - d-Glcp - (1 → and → 4,6) - d-Glcp - (1 →). The main chain connection of polysaccharides SGCP3 and NSGCP3 is → 4) - α - d-Glcp - (1 →, while the terminal group α - d-Glcp - (1 → is connected to the main chain through an O-6 bond.	UV, HPGPC, FT-IR, GC-MS, HPLC.	Antioxidant activity.	[67]
GPSa		4.23 × 10^5^ Da	Rha: Man: Glu = 1:1.07:67.24	The main structure of the sugar chain is α— (1→4) pyranose type d-glucose.	HPLC, IR, NMR.		[26]
YGEP AcYGEP				GEP is a pyranose, mainly d-glucose linked by alpha glycosidic bonds.	AF4-MALS-dRI,UV/Vis, FTIR, XRD, SEM.		[80]
GBI-1 GBI-2 GBI-3 GBII GBIII GBIV GBV		GBI-1: 14,600 GBI-2: 8700 GBI-3: 7000 GBII: 4300 GBIII: 19,000 GBIV: 87,000 GBV: 100,000			HPLC, GC-MS, FT-IR.	Treatment of acute liver injury.	[93]
GEP SeGEP	α			β–glycosidic bond linked polysaccharide structure with pyran ring as skeleton.	UV, FT-IR, HPLC, NMR, SEM.	Antioxidant activity.	[82]
PGE	α	1.54 × 10^3^ kDa	Glu	Alpha type d-glucose, with a pyran ring configuration, is mainly linked by glycosidic bonds with glucose as the main chain, and branches may include glucose with 1→3 linkage, glucose with 1→4→6 linkage, and glucose with 1 → linkage at the end.	HPLC, FT-IR, UV, NMR, ESM.		[86]
GeB40-1 GeB80-4	α	Greater than 8000 Da	GeB40-1:L-rhamnose:D-galactose:D-glucose:D-xylose:D-mannose = 29:1:42:11:2 GeB80-4:L-rhamnose:D-galactose:D-glucose = 2.5:1:25	α-glycosidic bond.	UV, HPLC, HPAEC.	Antibiotic activity.	[87]
WTM-2 WTM-3 WTM-5 WTM-6	α	WTM-2: 40.41 KDa WTM-3: 30.43 KDa WTM-5: 17.02 KDa WTM-6: 12.76 KDa	WTM-2:glucose:xylose:fructose:glucuronic acid = 72.43:4.12:1.00:0.24 WTM-3:glucose:xylose:fructose:glucuronic acid = 74.21:3.19:1.00:0.25 WTM-5:glucose:xylose:fructose:glucuronic acid = 70.07:5.13:1.00:0.34 WTM-6:glucose:xylose:fructose:glucuronic acid:mannose:= 69.62:0.57:1.00:0.35:0.24	(α-d-Glcp-(1→4)-α-d-Glcp)_n_.	HILIC-ELSD, UV, IR, NMR, SEM, XRD.	Antineoplastic activity.	[88]
WPGB-A-H	α	28,840 Da	Two kinds of monosaccharide composition of D-glucose and D-mannose.	α-type dextran, sugar ring configuration is pyran type.	HPLC, IR, UV, GC.		[89]
PRG1 PRG2 PRG3	α	PRG1: 19,251 Da PRG2: 3920 Da PRG3: 32,646 Da	PRG1: Fru: Glu = 1:10.682 PRG2: Glu PRG3: Fru: Glu = 1:1.542 PRG4: PRG5: Formed by dehydration condensation of glucose	The glycosidic bonds are all α-configuration.	HPGPC, HPLC, IR, UV.	Antioxidant activity.	[90]
TM1		2.02 × 10^5^ Da	Rha:Fuc:Xyl:Man:Glu = 0.22:14.80:0.36:39.00:66.52	It is composed of α (1-6)-d-Glc, containing a small amount of β-configuration, and the side chain branch point is at the C-4 position.	UV, GC, IR, NMR.		[92]

**Table 3:** Biological activities of *G. elata* polysaccharides and their underlying mechanisms of actions.

Biological Activities	Polysaccharide Name	CELL LINE	Modelling	In Vivo/In Vitro	Dosages	Mechanism of Action	Ref.
Anti-cancer activity							
pancreatic cancer	WTMA	PANC-1 and LO2	——	In vitro	——	Anti-cancer activity assay indicated that the polysaccharide could inhibit the growth of PANC- 1 cell.	[22]
Breast Cancer	GEP	MCF-7 cell	——	In vitro	——	The MCF-7 cell cycle was arrested at the S phase after AcGE treatment	[19]
antitumor	PGEs	MCF-7cell	——	In vitro	0, 1, 2, 4, 6 mg/mL	The PGEs can inhibit the growth of MCF-7 cells by inducing late apoptosis.	[107]
glioma resistance	PGEs	Rat glioma cells (C6)	——	In vivo	0.10, 0.25, 0.50, 1.00, 1.50 mg/mL	Inhibition of active migration in glioma cell growth.	[108]
pheochromocytoma (blood cell tumour)	PGEs	PC12 cell line	——	In vivo	0, 250, 500, 1000 μg/mL	Inhibits intracellular Ca^2+^ concentration overload, regulates the BCL-2/BAX ratio, and inhibits caspase pathways.	[79]
Transplantable tumours	PGEs	S180 sarcoma and H22 hepatocellular carcinoma strains	——	In vivo	0.8, 0.4, 0.2 g/kg	Destruction of tumour cell proliferation and enhancement of body immunity in hormonal mice, etc.	[109]
liver cancer	PGEs	Mouse Hepatocellular carcinoma H22 cell	——	In vivo	30, 60, 90 mg/kg	Affects the BCL-2/BAX-Caspase pathway in PC12 cells and regulates the mitochondrial apoptotic pathway.	[110]
loaded tumor	PGEs	H22 cell	——	In vivo	320, 160, 80 mg/kg	It affects cell cycle distribution, inhibits cell proliferation and activates the caspase system to induce apoptosis in tumour cells.	[111]
liver cancer	WSS25	RAW264.7 or BMM	RANKL treatment to induce osteoclastogenesis in mice	In vivo/in vitro	——	WSS25 inhibits RANKL-induced osteoclast formation in RAW264.7 cells and BMM by blocking the BMP-2/Smad/Id1 signalling pathway	[112]
colorectal cancer	PGEs	SW480 (Human colon cancer cell lines)	——	In vitro	500, 1000 μmol/mL	*G. elata* polysaccharide promotes the expression of miR-27a-3p and inhibits the expression of B7-H3	[46]
Immunomodulation							
Immunostimulatory Effect	PGEs	RAW264.7 Cells	Lipopolysaccharide inflammation model	In vitro	50, 100, 200 μg/mL	By activation of the NF-κB signaling pathway	[43]
Immnue-enhancing activity	PGEs	——	Intragastric administration of cyclophosphamide	In vivo	10 mL/kg	Improved the serum IL-2, TNF-a, IFN-g, IgG, IgA and IgM levels as well as the spleen and thymus indexes	[53]
immunomodulatory activity	PGEs	——	a mouse model of CTX-induced immunosuppression	In vivo	200, 400 mg/kg	The modulation of the com position of gut microbiota by GEPs and the resulting increased content of SCFAs.	[48]
Immunomodulatory Effects	SeGEP	RAW264.7 Cells	Cyclophosphamide-Treated Mice	In vivo/in vitro	5, 10, 15, 20 μg/mL	Se-GEP treatment enhanced cell viability and phagocytosis and increased the secretion levels of NO and TNF-α.	[50]
Neuroprotectiv effect	GEP	PC12 cell	A dose of 200 µmol/l CORT was selected to induce PC12 cell apoptosis	In vitro	250, 500, 1000 µg/mL	By inhibiting oxidative stress and ER stress-mediated apoptotic pathways.	[113]
low immune function	PGEs	——	CTX-induced immuno suppressive mouse model	In vivo	100, 200, 400 mg/kg	Significantly increases serum IgA, IgG, IgM and serum solubility. haemoglobin levels, increased spleen index and thymus index.	[114]
immune liver injury	PGE-2	——	Immune liver injury model induced by BCG + LPS in mice	In vivo	25, 50, 100 mg/kg	The hepatoprotective mechanism of GEP-2, may be through the production of homeostatic cytokines and the modulation of body immune function	[115]
Immunological Activity	RGP-1a and RGP-1b	RAW 264.7	cytotoxicity assay	In vitro	200 µg/mL	RGP-1a and RGP-1b significantly affected RAW 264.7 NO generation and phagocytic activity in a dose-dependent manner.	[55]
anti-ageing							
antioxidant activity analysis	PGEs	——	——	In vitro	1, 2.5, 4, 5.5, 6 mg/mL	The polysaccharide concentration was 1.0 mg/mL, the scavenging rates of DPPH and ABTS free radicals were both over 80%.	[35]
antioxidant	SeGEP	——	——	In vitro	10 mg/mL	DPPH free radical scavenging rate and ABTS free radical cation scavenging rate.	[82]
anti-ageing	PGEs	——	Aging model of mice injected with 5% D-galactose	In vivo	2.5, 5.0, 10.0 mg/(g.d)	SPGEs can improve the learning and memory ability of mice, DPPH free radical scavenging rate and ABTS free radical cation scavenging rate.	[116]
antioxidant	GEP-G GEP2 G	——	——	In vitro	1 mg/mL	Increased DPPH clearance, superoxide anion inhibition and hydroxyl radical inhibition.	[74]
antioxidant	PGEs	——	——	In vitro	0.5, 1.0, 1.5, 2.0, 2.5, 3.0, 3.5 mg/mL	Improve the scavenging efficiency of DPPH free radical and hydroxyl radical.	[62]
anti-ageing	PGEs	——	The aging mouse model was established by subcutaneous injection of 5% D-galactose (125 mg/kg).	In vivo	0.4, 2.0, 10.0 mg/(g·d^−1^)	Its mechanism may be related to the down-regulation of caspase-3, MAFbX and MURF-1 expression.	[117]
Antiviral/antibacterial/anti-inflammatory							
cerebral ischemia–reperfusion injury	NPGE	HT22 cell	mouse model of ischemic stroke (IS)	In vivo/in vitro	100, 250, 500 μg/mL	It inhibited neuroinflammation by down-regulating the level of IL-1β, IL-6, TNF-α, NLRP3, and HMGB1.	[118]
Akkermansia muciniphila	GEP-3, GEP-4	*Mucococcus* spp.	HFD and NASH mice	In vivo/in vitro	——	GEP-1 could promote the growth of A. muciniphila and L. paracasei strains.	[14,24]
Inflammatory bowel disease	GBP	——	dextran sulfate sodium (DSS)-induced IBD mice	In vitro	70 mg/kg	GBP treatment restores imbalances in the gut microbiota by increasing levels of beneficial bacteria.	[31]
Gut Microbiota	GEP-I	intestinal flora	simulated intestinal fluid (SIF),	In vitro	10 mg/mL	GEP-I promotes the growth of probiotics, inhibits the number of pathogenic bacteria, and significantly increases the concentration of acetic acid, etc., thus acting as a prebiotic.	[44]
anti-inflflammatory and neuroprotective effffects	GBP	——	Animal Model of Neuropathic Pain	In vivo	20 mg/kg	Suppression of neuroinflammation.	[99]
anti-dengue virus bioactivities	WGEW, AGEW	anti-dengue virus (Aedes albopictus C6/36 cells)	——	In vitro	——	WSS45 and WAS45 exhibited a protective effffect on dengue virus as indicated by the absence of CPE in in-fected C6/36 cells.	[23,56]
Dengue 2 virus	WSS45	BHK cells	dengue virus serotype 2 (DV2),	In vitro	——	WSS45 exerted potent inhibitory effect on DV2 through interfering with the interaction between viruses and targeted cells.	[119]
short-chain fatty acid	PGEB-3-H	intestinal flora	SD rats were experimental model.	In vivo	100, 200, 400 mg/(kg·d)	Medium and low dose additions of *G. elata* polysaccharides had a more pronounced effect on SCFA concentration.	[120]
Neuroprotection							
Neuroprotectiv effect	PGEs	PC12 cell	PC12 cell apoptosis induced by corticosterone (CORT)	In vitro	250, 500, 1000 mg/mL	Neuroprotective effects of GEP depend on inhibition of endoplasmic reticulum stress-mediated pathways.	[113]
neural protective	PGEs	——	The model was prepared by middle cerebral artery occlusion (MCAO)	In vivo	100 mg/kg	Reduction of cytokine TNF-α and IL-1β mRNA expression in the hippocampus and improvement of hippocampal neuronal function	[53]
antidepressant	PGEs	——	Lipopolysaccharide (LPS) -induced depressive mice	In vivo	——	GEPS improves survival from CORT-induced PC12 cell injury and reduces LDH release from damaged cells	[121]
nerve damage	PGEs	PC12 cell	Corticosterone-induced PC12 cell injury	In vitro	250, 500, 1000 μg/mL)	GEPS increased the survival rate of CORT-induced damaged PC12 cells and decreased the release rate of LDH.	[77]
cerebral palsy	PGEs	——	The cerebral palsy model was established by ligation of the left common carotid artery and hypoxia for 1 h in young rats.	In vivo	300, 150 mg/kg	The mechanism is related to the increase of NO, NE and 5-HT in the cerebral cortex and hippocampus, the decrease of ACHE level, the increase of eNOS expression and the protection of hippocampal tissues.	[122]
Glutamate damaged HT22 cells	PGEs	HT22 cell	HT22 cells were incubated with 50 μg·mL^−1^ glutamate for 6 h.	In vitro	12.5, 25, 50, 100, 200 μg/mL	The mechanism may be related to the up-regulation of HO-1 expression and improvement of cellular antioxidant capacity.	[123]
Cerebral ischemia	PGEs	——	The rat model of permanent focal cerebral ischemia was established by embolizing the right middle cerebral artery.	In vivo	100 mg/kg	The mechanisms may play a protective role for neurons in the ischemic region by promoting the proliferation of endogenous NSCs.	[124,125]
Brain nerve cells	PGEs	——	Young rat model of cerebral palsy.	In vivo	300 mg/kg	Significantly inhibited apoptosis in cerebral tissue of young rats with cerebral palsy	[126]
Cardiovascular system activity							
Spontaneously Hypertensive	Acidic Polysaccharides	——	Spontaneously hypertensive rats (SHR) fed with high fat diet.	In vivo	6 mg/kg	acidic polysaccharides from Gastrodia rhizome reduce hypertension and improve serum lipid levels.	[57]
Suppression of serum cholesterol	PGEs	——	rats fed a high-fat diet	In vivo	6 mg/kg	Significantly suppresses the risk of atherosclerosis by reducing serum total cholesterol and LDL levels in SD rats.	[127]
Non-alcoholic fatty liver disease	PGEs	——	High-fat diet induced model	In vivo	50, 100, 200 mg/kg	Inhibition of NF-κB, TNF-α, IL-1β, iNOS and COX-2 protein expression in liver tissue, and inhibition of Bax expression, up-regulation of Bcl-2 factor.	[76]
lowering of blood pressure	PGEs	——	Hypertensive rat model (RHR).	In vivo	50, 100, 200 mg/kg ·d	The mechanism of action is related to promoting the production of endogenous vasodilators and inhibiting the release of endogenous vasoconstrictors.	[128]
Other roles							
osteoclast differentiation	WSS25	RAW264.7 cells or mouse bone marrow macrophages (BMMs)	RAW264.7 cells or mouse bone marrow macrophages (BMMs) to induce osteoclastogenesis.	In vitro	2.5, 5, 10 µg/mL	WSS25 inhibits RANKL-induced osteoclast formation in RAW264.7 cells and BMMs by blocking the BMP-2/Smad/Id1 pathway.	[112]
anti-vertigo	PGEs	——	Dizziness in mice caused by mechanical rotation	In vitro	——	GEP and AMP were similar in activity, and both significantly reduced the time taken by stunned mice to escape from electric shock (*p* < 0.01) and increased the amount of food consumed by stunned mice.	[129]

## Data Availability

Not applicable.
